# Effect of Post‐Ablational Angiotensin Receptor‐Neprilysin Inhibitor on Atrial Fibrillation Recurrence: A Systematic Review and Meta‐Analysis in Asian Population

**DOI:** 10.1002/clc.70284

**Published:** 2026-04-06

**Authors:** Yangyang Wang, Wenjing Zhang, Yang Shao, Qiming Dong, Yunfei Gu, Hao Wang

**Affiliations:** ^1^ The Department of Cardiology Luoyang Central Hospital Affiliated to Zhengzhou University Luoyang Henan China; ^2^ The Department of Cardiology Sahlgrenska University Hospital Gothenburg Västra Götaland County Sweden; ^3^ Greater Baltimore Medical Center Towson Maryland USA

**Keywords:** atrial fibrillation, meta‐analysis, radiofrequency ablation, sacubitril/valsartan

## Abstract

**Background:**

The efficacy of angiotensin receptor‐neprilysin inhibitor (ARNI) in reducing recurrence risk of atrial fibrillation (AF) after ablation remains uncertain.

**Methods:**

We summarized the results about ARNI in preventing AF recurrence after radiofrequency ablation. After PROSPERO registration, two researchers independently screened the literature and assessed the risk of bias in the included studies. The fixed effects model was used to merge the effect sizes, and the results were reported separately depending on whether the study was a RCT or a cohort study and on the follow‐up duration. We further conducted subgroup analysis by comparing the effect of ARNI in reducing AF recurrence to that of angiotensin‐converting enzyme inhibitor and/or angiotensin receptor blocker (ACEI/ARB) and to that of the Blank group. Publication bias and sensitivity analysis were evaluated.

**Results:**

Both in RCTs and cohort studies, constant usage of ARNI for 6 and 12 months partly reduced the AF recurrence after ablation (using 3‐month ARNI in RCTs: RR = 0.56; 95% CI = 0.37–0.85; *p* = 0.006; using 6‐month ARNI in RCTs: RR = 0.57; 95% CI = 0.39–0.82; *p* = 0.003; using 12‐month ARNI in RCTs: RR = 0.65; 95% CI = 0.49–0.88; *p* = 0.005; using 6‐month ARNI in cohort studies: RR = 0.44; 95% CI = 0.32–0.61; *p* < 0.001). Subgroup analysis showed that, compared to ACEI/ARB or Blank, both in RCTs and cohort studies, ARNI was more effective in reducing the recurrence rate of atrial fibrillation after ablation.

**Conclusions:**

The using of ARNI may be effective in preventing the recurrence of AF after ablation, with ARNIs being shown to be more effective than ACEI/ARBs.

## Graphical abstract

In this meta‐analysis of randomized controlled trials and cohort studies comparing angiotensin receptor‐neprilysin inhibitor (ARNI), angiotensin‐converting enzyme inhibitor and/or angiotensin receptor blocker (ACEI/ARB), and no‐treatment control, the use of ARNI may be effective in preventing the recurrence of atrial fibrillation (AF) after ablation, with ARNIs demonstrating superior efficacy compared to ACEIs/ARBs.

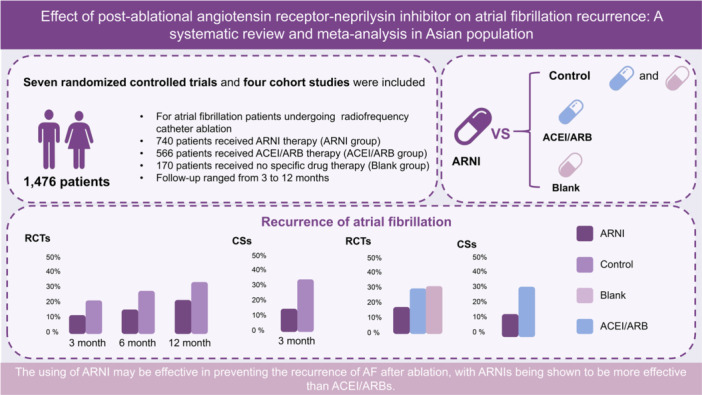

**Abbreviations**
AF: atrial fibrillation
ACEI/ARB: angiotensin‐converting enzyme inhibitor and/or angiotensin receptor blocker
ARNI: angiotensin receptor‐neprilysin inhibitor
Cis: confidence intervals
LAD: left atrial diameter
LVEF: left ventricular ejection fraction
RFCA: radiofrequency catheter ablation
RR: relative risk

## 1 Introduction

Atrial fibrillation (AF) is the most common type of cardiac arrhythmia [1], leading to serious complications, such as death, stroke, left ventricle dysfunction or heart failure (HF) [2, 3, 4]. Studies [5] have found that early rhythm control can effectively improve symptoms of atrial fibrillation, prevent its progression, and reduce the risk of cardiovascular complications. Among the strategies for rhythm control, radiofrequency catheter ablation (RFCA) has emerged as an effective treatment for symptomatic atrial fibrillation. However, the issue of post‐ablation recurrence continues to challenge electrophysiologists and limits patient prognosis. The recurrence rate of AF has been reported to be as high as 20% at 3 years and as high as 40% at 10 years [6]. Beyond diminishing quality of life, AF also imposes a significant economic burden on families and society. Therefore, the question of how to maintain sinus rhythm after catheter ablation in patients with AF over the long term warrants considerable attention.

The recurrence of atrial fibrillation is closely linked to atrial remodeling, with fibrosis being a fundamental aspect of this process [7]. Studies have shown that reversing left atrial remodeling may effectively improve the long‐term success rate of catheter ablation for persistent atrial fibrillation [8, 9]. Sacubitril‐valsartan, an angiotensin receptor‐neprilysin inhibitor (ARNI), has garnered increasing interest for the treatment of AF in recent years. Sacubitril‐valsartan inhibits atrial interstitial fibrosis induced by angiotensin‐II., atrial structural remodeling post‐fibrillation and prolonging the atrium's effective refractory period, which aids in preventing atrial fibrillation. Animal experiments have found that sacubitril‐valsartan can significantly reduce cardiac remodeling in animal models of AF [10]. While some research suggests that it may also be effective in preventing the recurrence of AF after RFCA, robust evidence‐based support is still lacking. Therefore, this meta‐analysis was conceived. We aim to summarize the evidence on ARNI's preventive effects on post‐ablation atrial fibrillation recurrence among the trials.

## 2 Methods

### 2.1 Data Sources and Study Identification

This study has been registered on the international prospective systematic review registration platform PROSPERO (website: https://www.crd.york.ac.uk/prospero/). The registration number is CRD42024570194. All randomized controlled trials (RCTs) and cohort studies published before July 6, 2024, that examined the effects of ARNI on patients undergoing ablation of AF were included. Studies were identified by searching the databases including PubMed, Embase, Web of Science and Cochrane Library, Database, China National Knowledge Network (CNKI), China Science and Technology Journal (CSTJ) Database and China Biomedical Database (CBM) using various combinations of terms, including “ARNI”, “Sacubitril‐valsartan”, “Angiotensin receptor neprilysin inhibitor” and “atrial fibrillation”. All studies retrieved were independently reviewed by two experienced researchers (YYW and HW) to minimize subjective selection bias. Studies were excluded if they met any of the following criteria: (i) review, meta‐analysis, conference abstract; (ii) animal experiments and mechanism studies; (iii) studies not in Chinese or English and (iv) studies with repetitive or unavailable data. Two investigators (YYW and HW) independently screened out studies that did not meet the inclusion criteria by reviewing titles and abstracts. The full texts were then thoroughly examined, and the literature was strictly screened according to the inclusion and exclusion criteria. Discrepancies were resolved through discussion or with the assistance of a third researcher (YFG). Data extraction included information on the number of patients, age, sex, history of hypertension, incidence of heart failure, duration and type of atrial fibrillation, radiofrequency ablation modality, follow‐up duration, event outcome, levels of N‐terminal prohormone of BNP (NT‐pro BNP), left ventricular ejection fraction (LVEF) and left atrial diameter (LAD). If specific data were not available, the corresponding authors were contacted via e‐mail or other methods.

### 2.2 Quality Assessment

The methodological quality was assessed using the Cochrane Risk of Bias Assessment Tool as implemented in Review Manager 5.3 software (Copenhagen: The Nordic Cochrane Centre, The Cochrane Collaboration, 2014). This tool evaluates seven domains: random sequence generation, allocation concealment, blinding of participants and personnel, blinding of outcome assessment, incomplete outcome data, selective reporting, and other sources of bias [11]. The Newcastle Ottawa Scale (NOS) was used to evaluate the quality of cohort studies [12, 13].

### 2.3 Data Analysis

Statistical analysis was conducted using ReviewManager5.3 (Copenhagen: The Nordic Cochrane Centre, The Cochrane Collaboration, 2014) and Stata18.0 (Stata, College Station, TX, USA). The follow‐up duration and treatment time for each included literature were found to be same. We divided the follow‐up time into 3 months, 6 months, and 12 months. The Outcome measures included both dichotomous variables. For dichotomous outcomes, relative risk (RR) with confidence intervals (CIs) was calculated. A P‐value of less than 0.05 was considered to indicate a statistically significant difference between the experimental and control groups. When *p* > 0.10 and *I* ^2^ < 50%, the heterogeneity between studies is small, and a fixed effects model is chosen for statistical analysis. When *p* < 0.10 and *I* ^2^ > 50%, statistical heterogeneity between different studies is significant, and a random effects model is selected for statistical analysis [14]. The results were reported separately according to the study design type (RCTs or cohort studies) and the follow‐up duration. We further conducted subgroup analysis according to the control group used in the included studies, angiotensin‐converting enzyme inhibitor and/or angiotensin receptor blocker (ACEI/ARB) or Blank. Publication bias was assessed using Begg's rank correlation and Egger's linear regression tests for parameters reported in fewer than 10 studies. If there is publication bias, then the trim‐and‐fill method was employed to correct for identified publication bias by conservatively imputing hypothetical negative unpublished studies before recalculating the meta‐analysis [15]. Sensitivity analysis and subgroup analysis were undertaken to investigate and mitigate sources of heterogeneity. Sensitivity analyses were conducted by excluding one study at a time using Stata version 18.0 (Stata, College Station, TX, USA).

## 3 Results

### 3.1 Characteristics of the Included Studies

**Figure 1:**
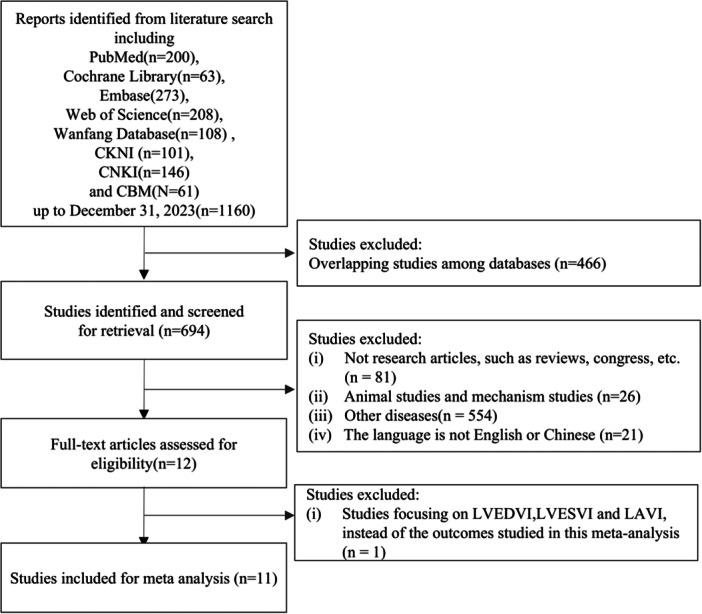
Flow diagram of studies included in this meta‐analysis.

Two independent researchers screened a total of 1160 potential studies. Among these studies, 466 were excluded due to duplication in databases, and 683 were eliminated after reviewing titles, abstracts, or full texts for relevance. Ultimately, 11 studies [16, 17, 18, 19, 20, 21, 22, 23, 24, 25, 26], all conducted in Asian populations, met the inclusion criteria and provided usable data (Figure 1). Table 1 displays the basic information of the included studies, and Table 2 outlines the characteristics of each study. The final analysis encompassed 1476 participants across the 11 studies: 740 were administered ARNI therapy (ARNI group), 566 received ACEI/ARB therapy (ACEI/ARB group), and 170 underwent without any ARNI/ACEI/ARB drugs (treated as the Blank group). Both the ACEI/ARB and the Blank groups were considered as the control groups in the present study. We finally included 7 RCTs [16, 17, 18, 19, 20, 21, 22] and 4 cohort studies [23, 24, 25, 26]. The results of the RCTs and cohort studies were presented separately. Four RCTs [17, 18, 19, 22] gave the ACEI/ARB treatment and therefore were considered as the control group. The other RCTs [16, 20, 21] were the Blank groups, which we also included as the control group. Four cohort studies [23, 24, 25, 26] were about the treatment of ACEI/ARB and considered as the control group. Three cohort studies [23, 24, 25] reported a 6‐month course.

**Table 1:** The Basic Information of the Study.

Author, year	Study type	AF type	No. Patients	Female	Follow‐up(months)	Ablation Type	Treatment Group	Control Group	Treatment Course (months)
Duan QQ 2022 [16]	RCT	Persistent	80	45	3, 6	RFCA	ARNI	Blank	6
Zang XB 2023 [17]	RCT	Persistent	130	53	12	RFCA	ARNI	ACEI/ARB	12
Yang L 2022 [18]	RCT	Paroxysmal/Persistent	64	29	6	RFCA	ARNI	ACEI/ARB	6
Wang QQ 2022 [19]	RCT	Persistent	138	44	12	RFCA	ARNI	ACEI/ARB	12
Yv P 2023 [20]	RCT	Persistent	80	29	3, 6	RFCA	ARNI	Blank	6
Huang Q 2021 [21]	RCT	Persistent	180	86	3, 6, 12	RFCA	ARNI	Blank	12
Li K 2022 [22]	RCT	Paroxysmal	100	40	3	RFCA	ARNI	ACEI/ARB	3
Dong YZ 2022 [23]	Prospective cohort	Paroxysmal/Persistent	300	126	7.6	RFCA	ARNI	ACEI/ARB	—
Liang XF 2023 [24]	Prospective cohort	Persistent	144	62	6	RFCA	ARNI	ACEI/ARB	6
Zhu TY 2022 [25]	Prospective cohort	Persistent	70	26	6	RFCA	ARNI	ACEI/ARB	6
Wang S 2023 [26]	Prospective cohort	Persistent	180	115	14.78 ± 3.67	RFCA	ARNI	ACEI/ARB	—

*Note:* The meta‐analysis ultimately included 11 studies conducted in Asian, comprising 7 randomized controlled trials (RCTs) and 4 prospective cohort studies, with a total of 1,476 patients. Detailed data as shown in the table.

Abbreviations: ACEI/ARB, angiotensin converting enzyme inhibitors/angiotensin receptor blockers; ARNI, angiotensin receptor‐neprilysin inhibitor; RCT, randomized controlled trial; RFCA, adiofrequency catheter ablation.

**Table 2:** The Characteristics of Each Study.

Author, year	Age (years)		Duration of AF (months)		Hypertension (%)		Heart failure (%)		NT‐proBNP (pg/mL)		LAD (mm)		LVEF (%)	
	ARNI Group	Control Group	ARNI Group	Control Group	ARNI Group	Control Group	ARNI Group	Control Group	ARNI Group	Control Group	ARNI Group	Control Group	ARNI Group	Control Group
RCT														
Duan QQ 2022 [16]	65.80 ± 7.81	63.68 ± 9.13	—	—	52.5	45	100	100	1817.43 ± 470.25	1779.00 ± 412.58	42.5 ± 2.8	42.2 ± 3.3	54.48 ± 1.74	54.03 ± 1.78
Zang XB 2023 [17]	63.25 ± 10.29	62.97 ± 11.16	35.92 ± 22.51	32.01 ± 21.39	100	100	29.9	36.5	768.22 ± 329.44	846.96 ± 1388.48	44.86 ± 6.08	45.40 ± 7.08	52.70 ± 9.12	53.24 ± 9.40
Yang L 2022 [18]	63.7 ± 9.0	64.8 ± 9.8	—	—	62.5	59.4	34.4	31.3	—	—	43 ± 5	43 ± 5	60.9 ± 6.6	60.6 ± 6.8
Wang QQ 2022 [19]	58.9 ± 12.75	62.7 ± 10.91	—	—	43.5	55.1	100	100	236 (168,320)	197 (136,278)	44 ± 6	44 ± 6	62(59.4,57.0)	62(59.2,66.0)
Yv P 2023 [20]	60.88 ± 8.77	62.78 ± 9.02	6.00 (1.00,23.75)	8.00 (2.00,45.00)	47.5	40. 0	—	—	—	—	48.50(43.25,51.75)	51.00 (43.25,54.00)	51.50(45.00,56.00)	50.00(48.25,54.00)
Huang Q 2021 [21]	67.5 ± 6.7	66.1 ± 7.9	19.2 ± 14.4	21.6 ± 10.8	72.2	70	100	100	1025.2 ± 363.7	993.2 ± 434.5	48.3 ± 2.5	47.1 ± 2.0	50.2 ± 4.8	48.5 ± 2.9
Li K 2022 [22]	61 ± 10	63 ± 9	—	—	56	70	100	100	781.00 (570.00,1147.00)	861.50 (681.00,1540.50)	39.06 ± 5.50	38.76 ± 3.91	60.50(58.00,65.00)	62.00(56.75,63.25)
Prospective cohort														
Dong YZ 2022 [23]	65.03(10.03)	65.82(9.63)	12.00 (3.00,45.00)	12.00 (2.00,36.00)	67.1	72.9	83.87	83.23	—	—	38.77(5.63)	38.92(5.14)	48.60(10.57)	50.28(9.03)
Liang XF 2023 [24]	70.08 ± 8.88	70.47 ± 6.90	70.47 ± 6.90	—	73.6	70.8	—	—	843.00(439.50,1502.50)	556.50(290.00,1410.00)	44.00 (41.25,48.00)	45.00 (42.25,48.00)	56.00(53.00,62.00)	57.50(48.50,62.00)
Zhu TY 2022 [25]	67.26 ± 11.97	65.2 ± 9.79	—	—	88.6	94.3	45.7	40	—	—	44.00 ± 6.83	43.40 ± 6.05	56.05 ± 13.37	64.28 ± 1.34
Wang S 2023 [26]	61.48 ± 10.01	62.07 ± 10.82	30(6,60)	24 (6.75,60)	55.6	60.0	55.6	51.1	—	—	42.74 ± 4.50	42.10 ± 4.66	57.76 ± 8.71	59.12 ± 8.50

*Note:* Data are presented as *n* (%), mean ± standard deviation, or median (interquartile range), as appropriate.

Abbreviations: AF, atrial fibrillation; ARNI, angiotensin receptor‐neprilysin inhibitor; LAD, left atrial diameter; LVEF, left ventricular ejection fraction; NT‐pro BNP, N‐terminal prohormone of BNP.

### 3.2 Literature Quality Assessment

**Figure 2:**
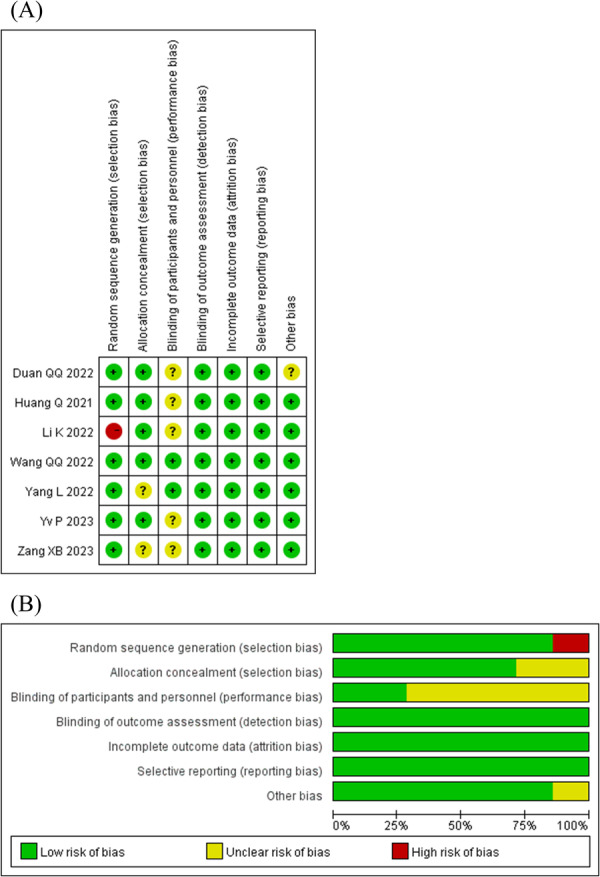
Risk of bias assessment for the included RCTs. (A) Summary of the risk of bias for each individual trial. (B) Overall risk of bias.

The risk of bias assessment for the RCTs is depicted in Figure 2. The quality assessment for the cohort studies is presented in Table 3, which includes the selection of the study population, the comparability between groups, and the measurement of outcomes, with a total of nine points. The quality of the included articles is relatively considerable.

**Table 3:** Results of Quality Assessment Using the Newcastle‐Ottawa Scale for Cohort Studies.

Study	①	②	③	④	⑤	⑥	⑦	⑧	Total scores
Dong YZ 2022[23]	1	1	1	0	1	1	1	1	7
Liang XF 2023[24]	1	1	1	1	1	1	1	1	8
Zhu TY 2022[25]	1	1	1	1	0	1	1	1	7
Wang S 2023[26]	1	1	1	0	1	1	1	1	7

*Note:* The Newcastle‐Ottawa Scale (NOS) for cohort studies was used for quality assessment. A study can be awarded a maximum of one score for each item, except for the item “Comparability of cohorts”, which allows a maximum of two scores. The total score ranges from 0 to 9, with a higher score indicating higher methodological quality.

Item descriptions: ① Representativeness of the exposed cohort; ② Selection of the non‐exposed cohort; ③ Ascertainment of exposure; ④ Demonstration that outcome of interest was not present at start of study; ⑤ Comparability of cohorts on the basis of the design or analysis; ⑥ Assessment of outcome; ⑦ Was follow‐up long enough for outcomes to occur; ⑧ Adequacy of follow up of cohorts.

### 3.3 Effects of ARNI on AF Recurrence

**Figure 3:**
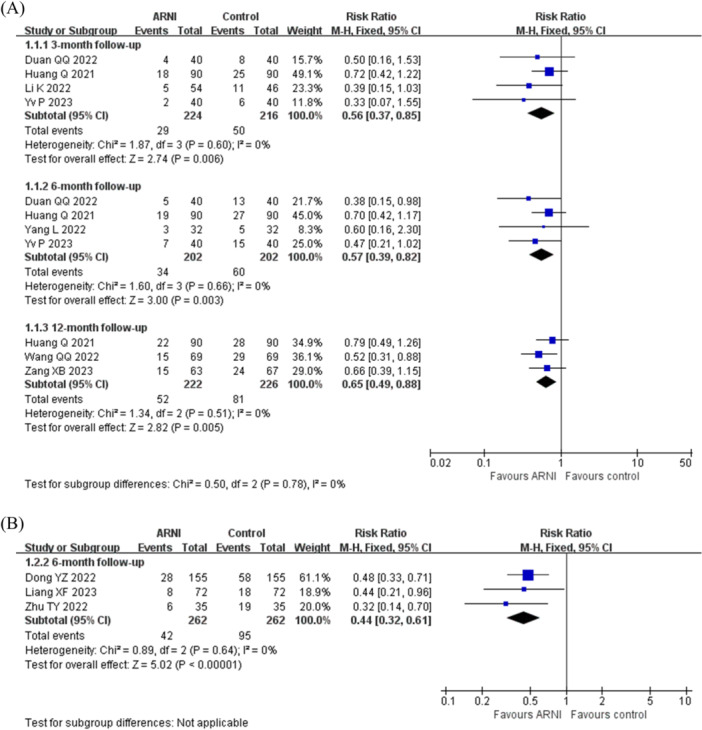
Forest plot of the meta‐analysis of different study types. (A) Forest plot with study type of RCT; (B) Forest plot with cohort study as the study type. The meta‐analysis at the 3‐month, 6‐month, and 12‐month follow‐ups demonstrated a consistently lower risk ratio (RR) for AF recurrence after RFCA in the ARNI group versus the control groups, with no significant heterogeneity observed.

In RCTs, the post‐ablation recurrence rate of AF in patients with continuous use of ARNI for 3 months was 12.9% (29 of 224 patients) compared to 23.1% (50 of 216 patients) of the control group, with a relative risk (RR) of 0.56 (95% CI = 0.37–0.85; *p* = 0.006; *I* ^2^ = 0%). The recurrence rate of AF post ablation in patients with the treatment of ARNI for 6 months after ablation was 16.8% (34 of 202 patients) versus 29.7% (60 of 202 patients) of the control group, with an RR of 0.57 (95% CI = 0.39–0.82; *p* = 0.003; *I* ^2^ = 0%). For 12 months of treatment of ARNI, the recurrence rate of AF post ablation was 23.4% (52 of 222 patients), in contrast to 35.8% (81 of 226 patients) of the control group, yielding an RR of 0.65 (95% CI = 0.49‐0.88; *p* = 0.005; *I* ^2^ = 0%), Figure 3A.

In the cohort studies, after 6 months of treatment with ARNI, the recurrence rate of AF post ablation was 16.0% (42 of 262 patients), compared to 36.3% (95 of 262 patients) of the control group, yielding an RR of 0.44 (95% CI = 0.32–0.61; *p* < 0.001; *I* ^2^ = 0%), Figure 3B.

### 3.4 Subgroup Analyses

**Figure 4:**
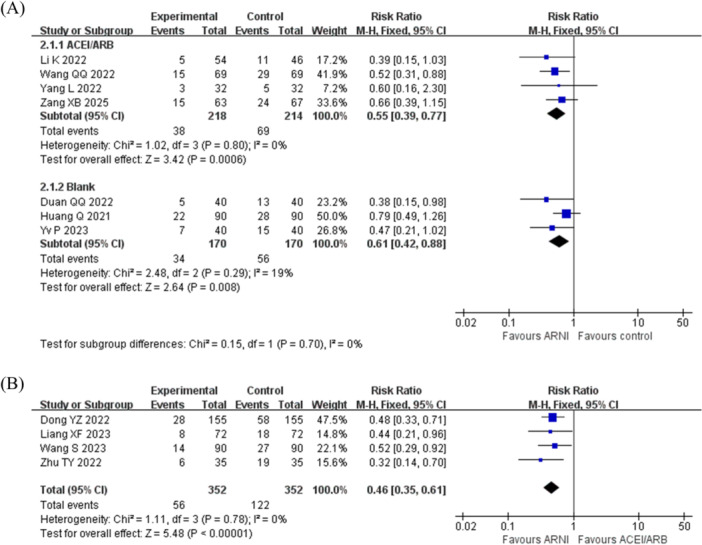
Forest plot of subgroup (according to different control groups) on recurrence. (A) In RCT, the analysis of ACEI/ARB or blank as the control group; (B) In the cohort studies, all control groups were analyzed using ACEI/ARB. The forest plot for the meta‐analysis of recurrence risk after radiofrequency catheter ablation of atrial fibrillation shows a significantly lower risk ratio (RR) in the ARNI group compared with the ACEI/ARB or blank groups, without significant heterogeneity.

Additionally, a separate subgroup analysis, stratified by comparison between ARNI and the control groups (the ACEI/ARB or the Blank group), was conducted. Compared to the ACEI/ARB control subgroup, the recurrence rate of AF post ablation in patients treated with ARNI was lower (in RCTs: RR = 0.55, 95% CI = 0.39–0.77, *p* < 0.001; in cohort studies: RR = 0.46, 95% CI = 0.35–0.61, *p* < 0.001) (Figure 4A,B). Similarly, compared to the Blank control subgroup, the recurrence rate of AF post ablation in patients treated with ARNI was lower (in RCTs: RR = 0.61, 95% CI = 0.42–0.88, *p* = 0.008) (Figure 4A). Furthermore, we further performed a subgroup analysis on studies that exclusively enrolled AF patients with concomitant HF. This analysis of four RCTs also demonstrated a significant reduction in AF recurrence with ARNI (RR = 0.57, 95% CI = 0.42–0.78, *p* < 0.001) (Figure S1).

### 3.5 Publication Bias

**Figure 5:**
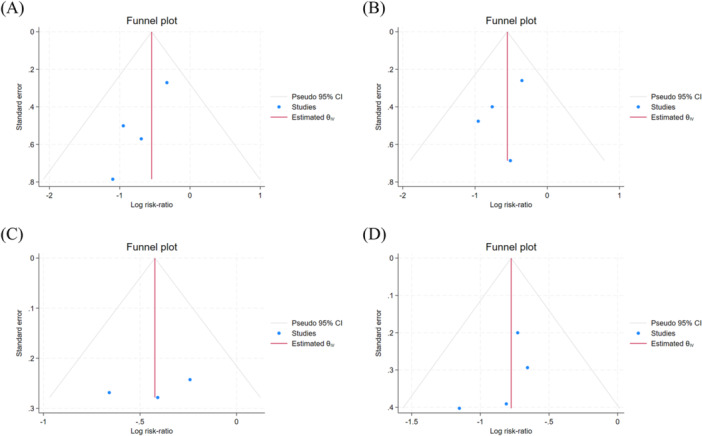
Funnel plots based on the study type. (A) Funnel plot of 3‐month follow‐up in RCTs; (B) Funnel plot of 6‐month follow‐up in RCTs; (C) Funnel plot of 12‐month follow‐up in RCTs; (D) Funnel plot of 6‐month follow‐up in cohort studies.

A visual inspection of funnel plots for study types revealed asymmetry (Figure 5). Given that each analysis included fewer than ten studies, we applied the Begg rank correlation test and the Egger linear regression test to evaluate publication bias. In RCTs, both tests indicated no significant publication bias for 3 months recurrence rates (Begg's test *z* = 0.34, *p* > 0.734; Egger's test *p* = 0.08), 6 months recurrence rates (Begg's test *z* = 0.34, *p* > 0.734; Egger's test *p* = 0.372) and 12 months recurrence rates (Begg's test *z* = 0.00, *p* > 1.000; Egger's test *p* = 0.539). In cohort studies, both tests indicated no significant publication bias for 6 months recurrence rates (Begg's test *z* = 1.02, *p* > 0.308; Egger's test *p* = 0.362). Given that the total number of studies was fewer than 20, and previous statistical analyses have demonstrated greater sensitivity of Egger's test compared to Begg's [27]. Therefore, based on Egger's test, there is no significant publication bias.

### 3.6 Sensitivity Analyses

**Figure 6:**
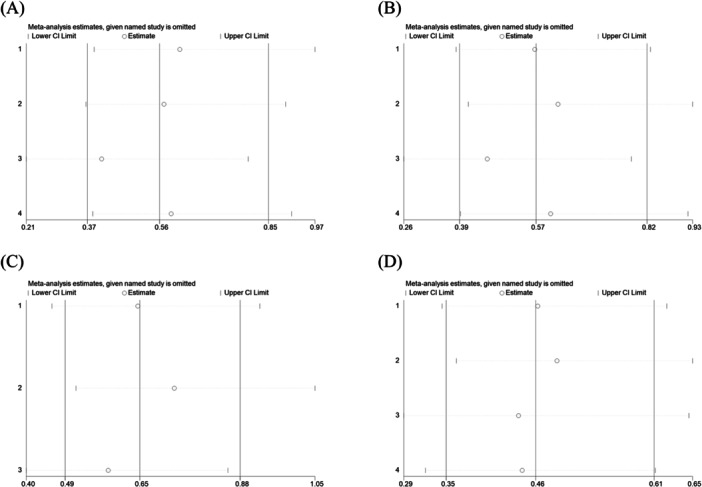
Sensitivity analysis based on the study type. (A) Sensitivity analysis of 3‐month follow‐up in RCTs; (B) Sensitivity analysis of 6‐month follow‐up in RCTs; (C) Sensitivity analysis of 12‐month follow‐up in RCTs; (D) Sensitivity analysis of 6‐month follow‐up in cohort studies. The significance of the pooled risk ratio did not change upon omission of any single study, confirming the stability of our finding that the ARNI group was associated with a significantly lower risk of recurrence.

Sensitivity analyses were conducted by excluding one study at a time using Stata version 18.0 (Stata, College Station, TX, USA). These analyses confirmed the robustness of the meta‐analytic results, as no significant changes were observed upon the removal of individual studies (refer to Figure 6), indicating that no single study unduly influenced the overall results.

### 3.7 Safety of ARNI on Observed Outcomes

Among the 11 studies, 5 studies reported safety data, of which 4 studies reported adverse events related to all cause hospitalization (3.91%), and 1 reported adverse events related to hypotension (5.56%), feeble (1.85%), gastrointestinal symptoms (1.85%), angioedema (1.85%), dizzy (3.70%), and worrisome of heart failure (2.78%). The relevant security data is shown in Table 4.

**Table 4:** Data on ARNI Security Reported in Studies.

		No. ARNI	All cause hospitalization	Hypotension	Feeble	Gastrointestinal symptoms	Angioedema	Dizzy	Worsening of heart failure
Duan QQ 2022 [16]		40	1	—	—	—	—	—	—
Yang L 2022 [18]		32	2	—	—	—	—	—	—
Li K 2022 [22]		54	—	3	1	1	1	2	—
Liang XF 2023 [24]		72	3	—	—	—	—	—	2
Zhu TY 2022 [25]		35	1	—	—	—	—	—	—

*Note:* Safety data were reported in five of the included studies. Data are expressed as number.

## 4 Discussion

This meta‐analysis is the first, to our knowledge, to report on the effects of angiotensin receptor‐neprilysin inhibitors (ARNI) on the recurrence of atrial fibrillation (AF) in patients post‐radiofrequency catheter ablation (RFCA). The findings suggest that ARNI may effectively prevent AF recurrence for at least 12 months post‐ablation. ARNI treatment has been shown to associate with a lower recurrence rate of AF post ablation than the blank (no treatment) and the angiotensin‐converting enzyme inhibitor/angiotensin receptor blocker (ACEI/ARB) groups. The present analysis showed relative low rate of adverse reactions of taking ARNI, less than 5.56%. We may expect an even lower rate in the actual cases due to the small sample size in the present meta‐analysis.

Atrial structural and electrical remodeling are critical in AF pathogenesis. Atrial fibrosis, a key indicator of structural remodeling [28], can be targeted by ARNI, which disrupts angiotensin II‐driven interstitial fibrosis, thereby mitigating structural remodeling and extending the atrial effective refractory period (AERP) [29]. This may contribute to reducing AF occurrence. ARNI also regulates matrix metalloproteinase (MMP) activity post‐AF, supporting cardiac structural integrity and enhancing myocardial pump function [30]. Recent studies have shown that sacubitril/valsartan, components of ARNI, can modulate calcium handling and reduce l‐type calcium current density, thus addressing electrical remodeling in AF. Furthermore, these agents have been found to decrease collagen deposition and fibrosis marker protein expression, improving atrial structural remodeling [31].

The additional benefit of ARNI over ARB may be due to the additive effect of sacubitril, a neprilysin inhibitor prodrug. Neprilysin degrades natriuretic peptides, which may modulate cardiac remodeling by preventing the fibrosis development. Research by Suo et al. [32] and Yang et al. [18] has shown that ARNI can improve atrial and appendage function more effectively than ARB alone. These benefits are also linked to the downregulation of fibrosis‐related pathways, including p‐Smad2/3, p‐p38 MAPK, and p‐JNK.

ARNI not only reverses atrial remodeling but also reduces atrial tension, as evidenced by decreased NT‐pro BNP levels. These effects have been observed in heart failure patients with reduced ejection fraction (HFrEF), where ARNI led to notable reductions in atrial and ventricular volume indices.

As a secondary study, this study cannot further identify which patients can reduce recurrence rates by using ARNI after ablation. Combining the protective mechanism of ARNI for AF patients, mapping the low voltage area within the atrium may be a useful identification method. This raises questions about whether endocardial mapping can be used to identify patients who would benefit most from ARNI. Our team's research suggests that sinus rhythm reflects the true low‐voltage areas, and mapping can be effectively performed after ablation to determine appropriate ARNI candidates, potentially reducing unnecessary use and healthcare costs. Consequently, we anticipate further studies in the future to validate this hypothesis.

### 4.1 Limitations

This study's findings are applicable primarily to the Asian population, as all the included trials were conducted in China. The small sample size underscores the need for further research to confirm these results more broadly. Additionally, several ongoing studies in the RCT network may provide more comprehensive data in the future. A limitation inherent in our meta‐analysis is the indirectness of results due to the absence of individual patient data, which precludes a more detailed analysis. Specifically, as the daily dose of ARNI varied among participants and detailed heart failure phenotypes (HFrEF, HFmrEF, HFpEF) were not consistently reported, we were unable to perform subgroup analyses based on dosage or HF subtype. Future individual patient data meta‐analyses are warranted to explore these important aspects. Notably, while ARNI has been observed to reduce left atrial size, a large left chamber does not uniformly correlate with poor voltage. Intracavitary mapping offers a direct measure of atrial size; however, none of the included studies provided such mapping data, contributing to indirect conclusions. Future trials that include mapping results are eagerly anticipated to enhance the directness of the evidence.

## 5 Conclusions

In conclusion, our meta‐analysis suggests that ARNI partly reduces the recurrence rate of AF following catheter ablation, with effects persisting for at least 12 months. Subgroup analyses for 3 months, 6 months, and 12 months follow‐up consistently indicate that ARNI is effective in reducing post‐ablation AF recurrence.

## Author Contributions

YYW and WJZ are responsible for the framework design of the entire meta‐analysis, the development of literature search strategies, the quality evaluation of screening studies, data extraction and integration, statistical analysis, as well as writing the initial and revised drafts of the paper. YS, QMD and YFG are responsible for guiding the use of data processing and statistical software. HW is responsible for reviewing the literature that is ultimately included in the analysis. Each author actively participates in various stages of the research, and through regular meetings and discussions, jointly solves problems that arise during the analysis process to ensure the quality of the research. All authors participated in the review and revision of the draft paper and agreed to the final submitted version.

## Ethics Statement

Ethical approval is not applicable to the meta‐analysis as it is a secondary study.

## Conflicts of Interest

The authors declare no conflicts of interest.

## Supporting information


**Figure S1:** Forest plot of the meta‐analysis comparing the effect of ARNI versus control on atrial fibrillation recurrence in patients with concomitant heart failure.

## Data Availability

Upon reasonable request, the corresponding author can provide the data that support the findings of this study (Hao Wang: wanghaoly@zzu.edu.cn).
