# Supplementary material for: Effect of Post‐Ablational Angiotensin Receptor‐Neprilysin Inhibitor on Atrial Fibrillation Recurrence: A Systematic Review and Meta‐Analysis in Asian Population
Source: Clin Cardiol. 2026 Apr 6;49(4):e70284. doi: 10.1002/clc.70284 (PMC13051897; doi:10.1002/clc.70284)
Supplement: Supplementary file 1 — Figure S1: Forest plot of the meta‐analysis comparing the effect of ARNI versus control on atrial fibrillation recurrence in patients with concomitant heart failure. [file CLC-49-e70284-s001.docx]

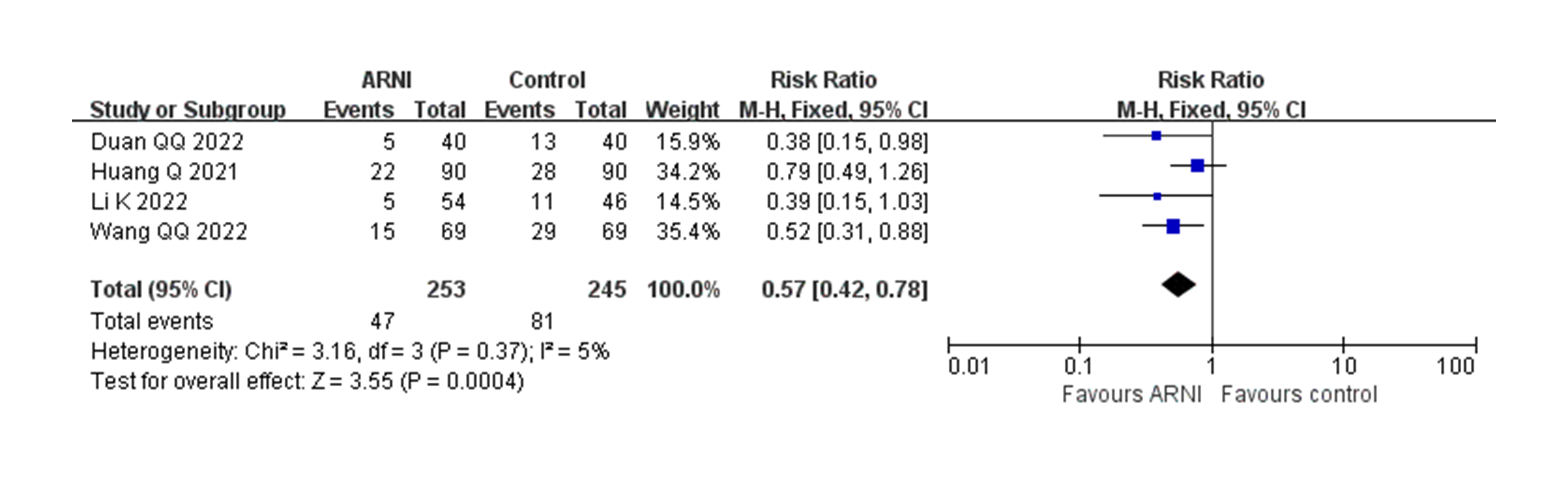


Figure S1 Forest plot of the meta-analysis comparing the effect of ARNI versus control on atrial fibrillation recurrence in patients with concomitant heart failure.
